# Prevalence of Subclinical Systemic Lymphedema in Patients Following Treatment for Breast Cancer and Association with Body Mass Index

**DOI:** 10.7759/cureus.7291

**Published:** 2020-03-16

**Authors:** Jose Maria Pereira de Godoy, Lívia Maria Pereira de Godoy, Maria de Fatima Guerreiro Godoy

**Affiliations:** 1 Cardiology and Cardiovascular Surgery, São José do Rio Preto School of Medicine (FAMERP), Sao Jose do Rio Preto, BRA; 2 Angiology and Vascular Surgery, The Godoy Clinic, Sao Jose do Rio Preto, BRA; 3 General Practice, The Godoy Clinic, São José do Rio Preto, BRA; 4 Medicine, São José do Rio Preto School of Medicine (FAMERP), São José do Rio Preto, BRA

**Keywords:** subclinical systemic lymphedema, treatment, breast cancer, body mass index

## Abstract

The aim of the present study is to evaluate the prevalence of subclinical systemic lymphedema in patients with lymphedema following treatment for breast cancer and the association with the body mass index (BMI). A cross-sectional study was conducted to determine the prevalence of subclinical systemic lymphedema using bioelectrical impedance analysis in patients with lymphedema following treatment for breast cancer. One hundred women with lymphedema of the upper limbs resulting from treatment for breast cancer were evaluated at the Godoy Clinic in 2019. Fisher's exact test demonstrated a significant association between BMI higher than 30 kg/m^2^ and subclinical systemic lymphedema (p=0.01). The prevalence of subclinical systemic lymphedema is high among patients with lymphedema following treatment for breast cancer and is associated with the increase in BMI.

## Introduction

Lymphedema of the upper limbs following treatment for breast cancer is highly prevalent in developed countries, affecting from 30% to 60% of patients submitted to the removal of axillary lymph nodes. Physiopathological processes, such as infection of the surgical wound, radiotherapy, obesity, and inadequate exercises/activities are aggravating factors associated with an increase in the prevalence of lymphedema and greater difficulty in terms of treatment [1-3].

Therapeutic techniques that affect physiopathological processes should be employed, such as those that lead to the significant mobilization of macromolecules. Manual lymphatic therapy adapted to physiopathology, mechanical lymphatic therapy, and compression mechanisms combined with lymphomyokinetic exercises or activities constitute the basis of treatment for lymphedema [4, 5]. The identification of risk factors and the establishment of preventive measures are fundamental to avoid the aggravation of lymphedema.

The diagnosis is performed based on measures of volume and circumference of the affected limb as well as bioelectrical impedance analysis. Volumetric analysis is the gold standard, but bioimpedance devices that measure water content in different parts of the body are quite useful due that the possibility of repeating the analysis as many times as necessary. The determination of circumferences is the most widely used exam due to the low cost and ease of use. However, there is no well-established prophylactic approach and each service uses what is considered to be the most suitable [5, 6].

Obesity is an aggravating factor of both lymphedema and venous disease and can be an isolated cause of edema [7, 8]. Therefore, obesity constitutes a physiopathological factor to consider in patients with lymphedema following treatment for breast cancer.

The aim of the present study is to evaluate the prevalence of subclinical systemic lymphedema in patients with lymphedema following treatment for breast cancer and the association with the body mass index (BMI).

## Materials and methods

One hundred women with lymphedema of the upper limbs resulting from treatment for breast cancer were evaluated at the Godoy Clinic in 2019. A cross-sectional study was conducted to determine the prevalence of subclinical systemic lymphedema using bioelectrical impedance analysis (InBody S10 analyzer; InBody Co., Seoul, Korea) in patients with lymphedema following treatment for breast cancer. Patients with a history of treatment for breast cancer who developed lymphedema of the upper limbs were included in the study. Patients with other causes of clinically diagnosed edema not associated with the treatment of breast cancer were excluded from the study. Patients were selected consecutively until reaching 100 participants.

Descriptive statistics were performed for the determination of prevalence values. Fisher's exact test was used for comparisons between groups, considering an alpha error of 5%. This study received approval from the human research ethics committee of the São José do Rio Preto School of Medicine (FAMERP) # 3.503.675.

## Results

One hundred consecutive patients were evaluated. Their age ranged from 32 to 80 years (mean: 61.01 years; median: 62 years). BMI ranged from 21 to 43.4 kg/m^2^ (mean: 31.01 kg/m^2^). Fourteen patients had a BMI of less than 25 kg/m^2^, 35 patients had a BMI between 25 and 30kg/m^2^, and 51 had a BMI higher than 30 kg/m^2^. None of the 14 patients with a BMI of less than 25 kg/m^2^ had subclinical systemic lymphedema. In contrast, six of the 35 patients with BMI between 25 and 30 kg/m^2^ and 20 of the 51 patients with a BMI higher than 30 kg/m^2^ had subclinical systemic lymphedema (see Table 1). Fisher's exact test demonstrated a significant association between BMI higher than 30 kg/m^2^ and subclinical systemic lymphedema (p=0.01).

**Table 1:** Prevalence subclinical systemic lymphedema and BMI range BMI - body mass index

	Patients	Subclinical systemic lymphedema	Prevalence (%)
BMI < 25	14	0	0
BMI 25 to 30	35	6	17.14
BMI > 30	51	20	39.21

Thirteen (26.53%) of the 49 patients with BMI < 30 kg/m^2^ and 20 (39.21%) of the 51 patients with BMI > 30 kg/m^2 ^had bilateral lymphedema of the upper limbs. However, this difference did not achieve statistical significance (Fisher's exact test, p=0.1). Regarding subclinical systemic lymphedema, 18 (36.73%) of the 49 patients with BMI < 30 kg/m^2^ and 46 (90.01%) of the 51 patients with BMI > 30 kg/m^2^ had edema of the trunk. This difference was statistically significant (Fisher's exact test, p=0.001).

## Discussion

The present study shows that patients with lymphedema following treatment for breast cancer can exhibit an increase in body liquid that involves the entire body. Current bioimpedance devices enable the analysis of liquids in the trunk and limbs.

A novel concept of lymphedema, denominated subclinical systemic lymphedema, has recently been described and is characterized by the increase in the intracellular and extracellular liquid above normal values in the trunk and limbs [9, 10]. This abnormality has been seen in patients over the years. However, obesity studies using animal models have revealed that the increase in weight is accompanied by changes in the lymphatic pump, capillary permeability, and the inflammatory process [9]. The generalized edema found with the increase in BMI in the present investigation is compatible to that seen in animal studies. Therefore, an increase in BMI constitutes another physiopathological mechanism of lymphedema. Indeed, weight loss is associated with an improvement in lymphedema [5]. 

Bioelectrical impedance analysis enables the identification of body liquids, the determination of edema in the trunk and limbs, and the monitoring of the dynamics of body liquids over time [9-11]. Normally, the lower limbs and the arm with lymphedema are the first to swell. In the present study, edema was associated with BMI, as none of the patients with BMI less than 25 kg/m^2^ had lymphedema, whereas the occurrence of this type of edema increased significantly with the increase in BMI.

Such findings indicate a new line of research addressing the physiopathological processes of edema in these patients. Each additional physiopathological process increases the probability of lymphedema, increasing the difficulty of treatment, and the maintenance of the results. More than half of these patients have edema throughout the body beyond the arm affected by surgery.

## Conclusions

The prevalence of subclinical systemic lymphedema is high among patients with lymphedema following treatment for breast cancer and is associated with the increase in BMI.
